# Neutralizing Antibody Responses Induced by HIV-1 Envelope Glycoprotein SOSIP Trimers Derived from Elite Neutralizers

**DOI:** 10.1128/JVI.01214-20

**Published:** 2020-11-23

**Authors:** Anna Schorcht, Tom L. G. M. van den Kerkhof, Christopher A. Cottrell, Joel D. Allen, Jonathan L. Torres, Anna-Janina Behrens, Edith E. Schermer, Judith A. Burger, Steven W. de Taeye, Alba Torrents de la Peña, Ilja Bontjer, Stephanie Gumbs, Gabriel Ozorowski, Celia C. LaBranche, Natalia de Val, Anila Yasmeen, Per Johan Klasse, David C. Montefiori, John P. Moore, Hanneke Schuitemaker, Max Crispin, Marit J. van Gils, Andrew B. Ward, Rogier W. Sanders

**Affiliations:** a Department of Medical Microbiology, Amsterdam Infection & Immunity Institute (AI&II), Amsterdam UMC, University of Amsterdam, Amsterdam, the Netherlands; b Department of Experimental Immunology, Amsterdam Infection & Immunity Institute (AI&II), Amsterdam UMC, University of Amsterdam, Amsterdam, the Netherlands; c Department of Integrative Structural and Computational Biology, The Scripps Research Institute, La Jolla, California, USA; d School of Biological Science, University of Southampton, Southampton, United Kingdom; e Department of Surgery, Duke University Medical Center, Durham, North Carolina, USA; f Department of Microbiology and Immunology, Weill Medical College of Cornell University, New York, New York, USA; g Janssen Pharmaceuticals, Leiden, the Netherlands; h Center for Molecular Microscopy, Center for Cancer Research, National Cancer Institute, National Institutes of Health, Frederick National Laboratory, Leidos Biomedical Research Inc., Frederick, Maryland, USA; Emory University

**Keywords:** Env trimer, SOSIP trimer, elite neutralizer, immunogenicity, neutralizing antibodies

## Abstract

The induction of broadly neutralizing antibodies (bNAbs) is a major goal in vaccine research. HIV-1-infected individuals that develop exceptionally strong bNAb responses, termed elite neutralizers, can inform vaccine design by providing blueprints for the induction of similar bNAb responses. We describe a new recombinant native-like envelope glycoprotein (Env) SOSIP trimer, termed AMC009, based on the viral founder sequences of an elite neutralizer. The subtype B AMC009 SOSIP protein formed stable native-like trimers that displayed multiple bNAb epitopes. Overall, its structure at 4.3-Å resolution was similar to that of BG505 SOSIP.664. The AMC009 trimer resembled one from a second elite neutralizer, AMC011, in having a dense and complete glycan shield. When tested as immunogens in rabbits, the AMC009 trimers did not induce autologous neutralizing antibody (NAb) responses efficiently while the AMC011 trimers did so very weakly, outcomes that may reflect the completeness of their glycan shields. The AMC011 trimer induced antibodies that occasionally cross-neutralized heterologous tier 2 viruses, sometimes at high titer. Cross-neutralizing antibodies were more frequently elicited by a trivalent combination of AMC008, AMC009, and AMC011 trimers, all derived from subtype B viruses. Each of these three individual trimers could deplete the NAb activity from the rabbit sera. Mapping the polyclonal sera by electron microscopy revealed that antibodies of multiple specificities could bind to sites on both autologous and heterologous trimers. These results advance our understanding of how to use Env trimers in multivalent vaccination regimens and the immunogenicity of trimers derived from elite neutralizers.

**IMPORTANCE** Elite neutralizers, i.e., individuals who developed unusually broad and potent neutralizing antibody responses, might serve as blueprints for HIV-1 vaccine design. Here, we studied the immunogenicity of native-like recombinant envelope glycoprotein (Env) trimers based on viral sequences from elite neutralizers. While immunization with single trimers from elite neutralization did not recapitulate the breadth and potency of neutralization observed in these infected individuals, a combination of three subtype B Env trimers from elite neutralizers resulted in some neutralization breadth within subtype B viruses. These results should guide future efforts to design vaccines to induce broadly neutralizing antibodies.

## Abstract (precis)

Elite neutralizers, i.e., individuals who developed unusually broad and potent neutralizing antibody responses, might serve as blueprints for HIV-1 vaccine design. Here, we studied the immunogenicity of native-like recombinant envelope glycoprotein (Env) trimers based on viral sequences from elite neutralizers. While immunization with single trimers from elite neutralization did not recapitulate the breadth and potency of neutralization observed in these infected individuals, a combination of three subtype B Env trimers from elite neutralizers resulted in some neutralization breadth within subtype B viruses. These results should guide future efforts to design vaccines to induce broadly neutralizing antibodies.

## INTRODUCTION

A successful human immunodeficiency virus type 1 (HIV-1) vaccine should be able to protect against HIV-1 acquisition, which probably requires the induction of high-titer, broadly neutralizing antibodies (bNAbs) (1, 2). Although this goal is challenging, two observations in humans support the pursuit of bNAb-inducing vaccines. First, B cells from some HIV-1-infected individuals are able to produce bNAbs (3–8). Elite neutralizers, the top 1% in study cohorts, develop exceptionally broad and potent antibody responses, even as soon as 10 months postseroconversion (post-SC) (9). Such individuals may serve as blueprints for vaccine designs aimed at inducing similar bNAb responses. Second, passive immunization of nonhuman primates (NHPs) with bNAbs, even at low doses, can block infection after repeated simian-human immunodeficiency virus (SHIV) challenges (10–15). A neutralization titer (50% infective dose [ID_50_]) in the range 1:100 to 1:500 may be protective against SHIV acquisition (16, 17). Candidate HIV-1 vaccines based on the SOSIP design have induced autologous and, occasionally, heterologous neutralizing antibody (NAb) titers in both rabbits and macaques (18–20). However, to date, no immunogen has elicited antibodies that strongly and consistently counter a range of diverse neutralization-resistant (i.e., tier 2) viruses.

The HIV-1 envelope glycoprotein (Env) is a heterotrimeric, labile complex comprising three gp120 and three gp41 subunits that are noncovalently associated and embedded in the viral membrane. The fragility of the intersubunit interactions hindered vaccine design for many years, but stable native-like trimers, such as ones based on the SOSIP design, now can be made for many Env genotypes. A series of modifications over a multiyear period improve SOSIP trimer formation and stability, reduce exposure of non-NAb V3 and CD4i epitopes, and allow presentation on nanoparticles (21–25). Alternative trimer designs have also been reported (26–29).

Attempts to broaden the NAb responses have included evaluating sequential and combination immunization regimens using SOSIP trimers derived from different HIV-1 subtypes (30, 31). While bNAbs were not induced, some useful information emerged. Klasse et al. showed that the autologous NAb response to a trimer combination was focused on shared holes in the glycan shield (30). Torrents de la Peña et al. reported that SOSIP trimers from different subtypes are too antigenically distinct and, hence, induce independent autologous NAb responses, rather than more cross-reactive NAbs, when tested together (31). An inference is that combining more closely related SOSIP trimers, such as ones derived from the same subtype, with a dense glycan shield might focus the immune response toward less immunogenic but more conserved epitopes, eventually resulting in a NAb response recognizing shared epitopes on different viral strains (32).

Here, we describe the generation, characterization, structure, and immunogenicity of a new SOSIP trimer based on early *env* sequences from AMC009, an elite neutralizer. This trimer then was combined with two others, AMC011 and AMC008, to create a trivalent immunogen. These three trimers are all based on subtype B sequences, allowing us to assess whether their antigenic similarity is beneficial for the induction of cross-reactive antibodies against shared epitopes. We found that the trivalent combination, in contrast to the AMC009 trimer alone, elicited antibodies that targeted different regions on SOSIP trimers and that occasionally cross-neutralized heterologous tier 2 viruses (19, 33).

## RESULTS

### Stable native-like Env trimers based on early viral sequences from elite neutralizers.

**FIG 1:**
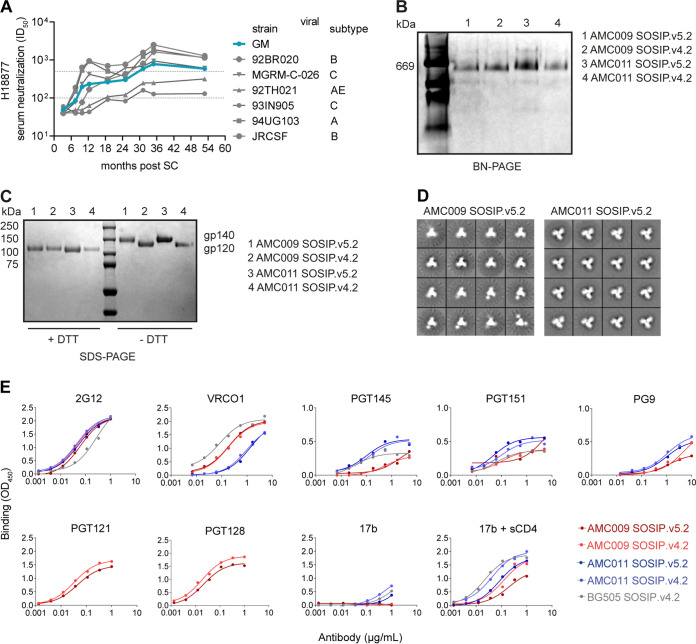
Characterization of the AMC009 and AMC011 SOSIP trimers. (A) Longitudinal sera from individual H18877 (source of AMC009 virus) were tested for neutralization activity against a 6-virus panel that represents globally circulating HIV-1 strains (8). The geometric mean (GM) ID_50_ of the 6 viruses is represented by the turquoise line. Based on the GM reaching 1:100 and 1:500 (indicated by the dotted lines), H18877 is classified as a broad neutralizer at ∼10 months and as an elite neutralizer at ∼30 months post-SC. (B and C) The AMC009 and AMC011 SOSIP.v4.2 and SOSIP.v5.2 trimers were expressed in 293F cells and PGT145 purified. (B) Analysis of the SOSIP.v4.2 and SOSIP.v5.2 variants by BN-PAGE. (C) SDS-PAGE of the AMC009 and AMC011 SOSIP.v4.2 and SOSIP.v5.2 trimers under both reducing (with dithiothreitol [+DTT]) and nonreducing (−DTT) conditions. Cleaved (gp120) and uncleaved (gp140) bands are indicated. (D) NS-EM 2D class averages of AMC009 and AMC011 SOSIP.v5.2 trimers (Table 2). (E) D7324 capture ELISA showing binding of bNAbs and non-NAb 17b, with or without sCD4, to AMC009 and AMC011 SOSIP.v4.2 and v5.2 trimers and to BG505 SOSIP.v4.2 for comparison. The *y* axis was adjusted for PGT145, PGT151, and PG9 and reflects the binding stoichiometry. OD_450_, optical density at 450 nm.

The AMC009 *env* gene was derived from HIV-1 (subtype B)-infected individual H18877, participating in the Amsterdam Cohort Studies on HIV/AIDS (ACS), who was classified as an elite neutralizer (34). The *env* genes from five viral biological clones isolated 2 months post-SC were then used to generate a consensus sequence that has only minor differences in the *env* variable regions compared to each clone. The biological clone 1.B5 was used to generate the AMC009 virus, which was categorized as a tier 2 virus (Table 1). The rationale for using early sequences was based on the hypothesis that bNAb development is initiated by epitopes exposed on early viruses (35). At month 2 pSC, serum from individual H18877 neutralized a panel of viruses, reflecting global diversity, with a geometric mean (GM) ID_50_ of 50 (cutoff, ID_50_ of 40) (Fig. 1A) (8). The NAb breadth and potency increased over time, and cross-subtype NAbs were already present at ∼10 months post-SC (8). Serum from month 31 neutralized the virus panel with a GM ID_50_ of 782, classifying H18877 as an elite neutralizer (34) (Fig. 1A). The target(s) for the neutralizing activity present in this individual’s sera has not been identified.

**TABLE 1:**
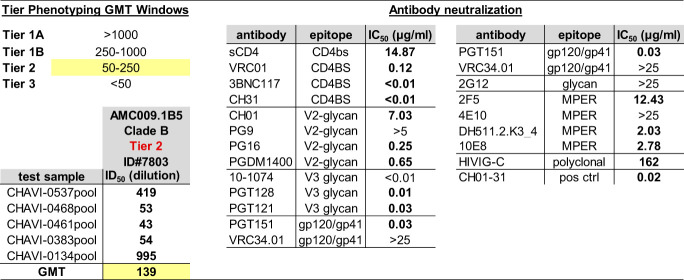
Neutralization tier classification of the AMC009 virus

To determine the tier, the neutralization sensitivity of the virus against serum pools and its sensitivity to well-characterized antibodies were assessed in a standard TZM-bl cell assay (see Materials and Methods). The targeted epitope of the tested bNAbs are listed. The indicated values are the plasma dilution (or antibody concentration, in μg/ml, for the bNAbs) at which the relative luminescence units (RLUs) were reduced 50% compared to those of the virus control wells.

Our initial AMC009 consensus gp140 applied the SOSIP.664 design, but when trimer design improvements became available, they were used to create the AMC009 SOSIP.v4.2 and SOSIP.v5.2 variants (18, 19, 29, 36). These sequential modifications improved trimer yields, stability, and antigenicity (Table 2).

**TABLE 2:** Biophysical properties of AMC009 and AMC011 SOSIP trimers

Parameter	Value by Env trimer and SOSIP version^*c*^			
	AMC009		AMC011	
	4.2	5.2	4.2	5.2
Production^*a*^ (yield [mg/liter])	1.6^*b*^	1.52^*b*^	4.75	2.1
Morphology by NS-EM (% native-like trimers)	100^*b*^	100	ND	100
Thermostability by DSC (two-state model [*T_m_* in °C])	68	70	63	67
Glycan composition by HILIC-UPLC (%)				
Man_5–9_	72	77*^b^	72	78*
Man_9_	34	32*^b^	37	32*

a Results were obtained from 293F cell-expressed and PGT145-purified SOSIP trimers.

b Results were obtained with D7324-tagged proteins.

c ND, not determined; *, quantified without Endo H digestion.

In addition, further stabilizing mutations were introduced to AMC011 SOSIP.v4.2 (9, 33), which is based on *env* genes derived from another elite neutralizer of the ACS, resulting in the SOSIP.v5.2 variant.

PGT145-purified AMC009 and AMC011 SOSIP variants yielded trimers (Fig. 1B) that were completely cleaved between gp120 and gp41 (Fig. 1C). We used negative-stain electron microscopy (NS-EM) to study the morphology of purified AMC009 SOSIP trimer variants and the AMC011 SOSIP.v5.2 variant. The reference-free two-dimensional (2D) class averages revealed that ∼100% of these proteins adopted a native-like trimer morphology (Fig. 1D and Table 2). Differential scanning calorimetry (DSC) experiments showed that the midpoint of thermal denaturation (*T_m_*) of AMC009 SOSIP.v4.2 was 68°C (Table 2), which is comparable to that of BG505 SOSIP.664 (*T_m_* = 68°C) (18). The additional disulfide bridge between residues 73C and 561C, present in the SOSIP.v5.2 design, increased the thermostability of the AMC009 trimer by 2°C (*T_m_* = 70°C) and by 4°C for the AMC011 trimer (*T_m_* = 67°C for SOSIP.v5.2 versus 63°C for SOSIP.v4.2) (33).

To study the antigenicity of the AMC009 and AMC011 SOSIP.v4.2 and SOSIP.v5.2 trimers, we used an enzyme-linked immunosorbent assay (ELISA) and a panel of bNAbs and non-NAbs (Fig. 1E). The AMC009 SOSIP.v4.2 trimer engages with bNAbs 2G12, VRC01, PGT121, and PGT128 as well as with quaternary structure-preferring bNAbs PGT145, PGT151, and PG9. The BG505 SOSIP.v4.2 trimer reduced binding of 17b, also in the presence of soluble CD4. The 73C-561C disulfide bond present in the AMC009 and AMC011 SOSIP.v5.2 trimers had no effect on the binding of bNAbs 2G12, VRC01, PGT145, and PGT151 and a panel of nonneutralizing antibodies, unlike the corresponding v4.2 variant. The non-NAb 17b bound less well to the SOSIP.v5.2 variants, both in the absence and presence of soluble CD4. There was a slight decrease in binding of PGT128 and PGT121 to AMC009 SOSIP.v5.2. Overall, the SOSIP.v5.2 changes had no major impact on antigenicity compared to SOSIP.v4.2, which is in line with previous observations with BG505 SOSIP trimer variants (29).

We conclude that the AMC009 SOSIP trimers are stable, have a native-like morphology, and present desired bNAb epitopes appropriately. The SOSIP.v5.2 variants of AMC009 and AMC011 were more thermally stable than SOSIP.v4.2, consistent with what has been reported previously for other trimer genotypes (29).

### Structural similarity of AMC009 SOSIP.v4.2 and BG505 SOSIP.664.

**FIG 2:**
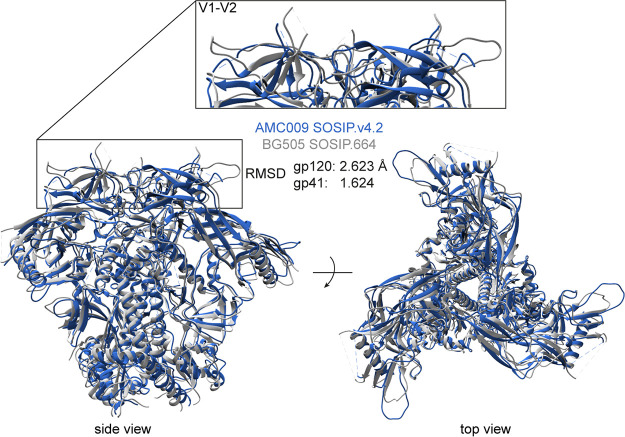
Overlay of the 3D cryo-EM reconstruction of AMC009 SOSIP.v4.2 trimers at a resolution of 4.3 Å (in blue) with the BG505 SOSIP.664 structure (PDB entry 4TVP; in gray) (37). The RMSD (in Å) for gp120 and gp41 are indicated. The inset shows the V1/V2 region.

**FIG 3:**
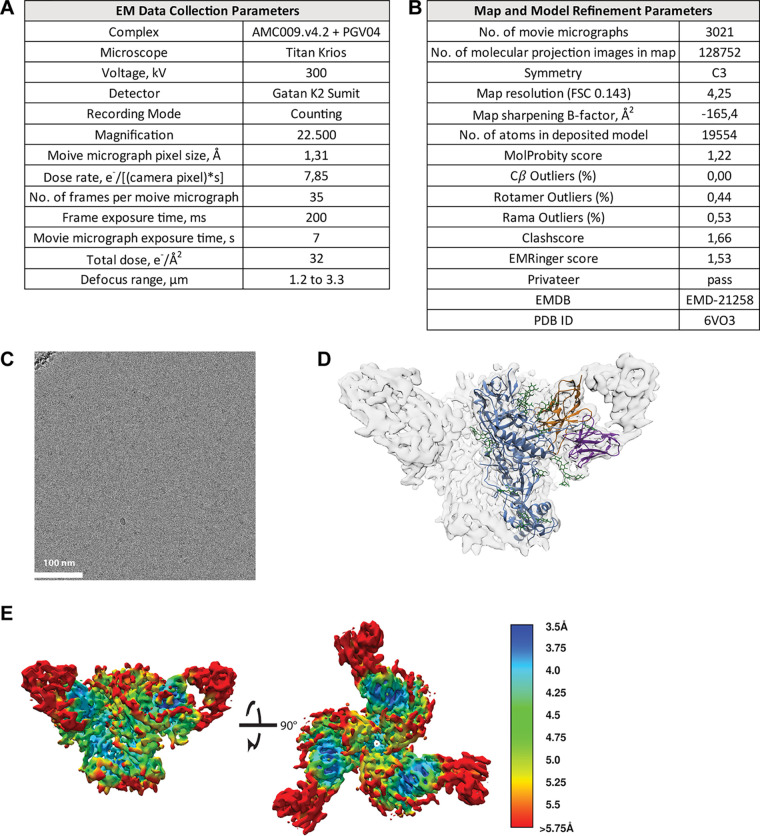
Cryo-EM parameters used to generate Fig. 2. (A) Parameters for data collection. (B) Modeling parameters of the AMC009 SOSIP.v4.2 trimer in complex with PGV04 Fab (84). (C) Representative micrograph for cryo-EM data set. (D) EM density map (transparent gray) for the AMC009 SOSIP.v4.2 trimer/PGV04 Fab atomic model. The trimer is shown in blue, N-linked glycans are in green, the PGV04 Fv heavy chain is in orange, and the PGV04 Fv light chain is in purple. (E) The local resolution of the AMC009 SOSIP.v4.2 structure is shown. Colors indicate the obtained resolution, ranging from 3.5 Å (dark blue) to >5.75 Å (red). The color legend is depicted on the right.

**FIG 4:**
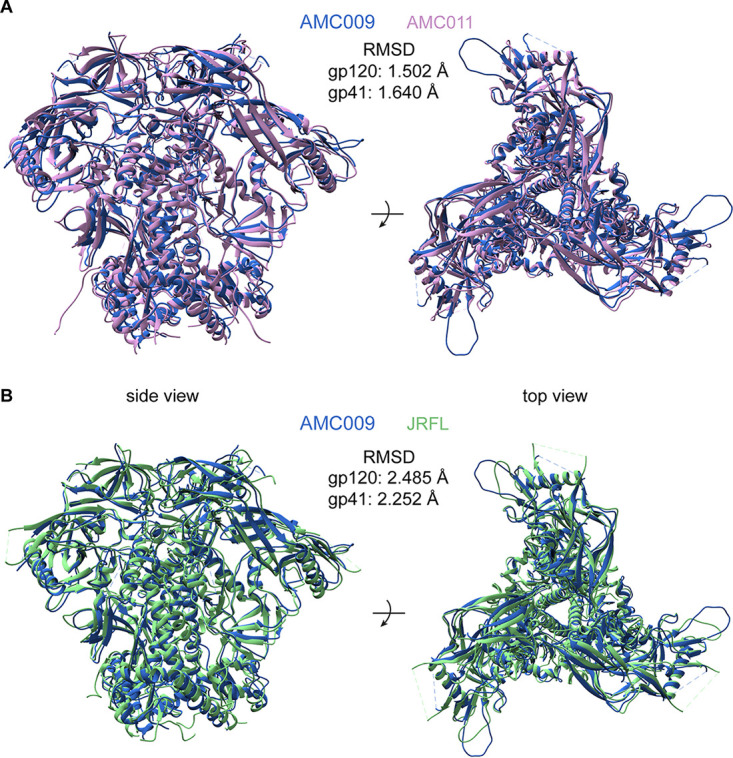
Overlay of the AMC009 SOSIP.v4.2 cryo-EM structure (PDB entry 6VO3; blue). (A) AMC011 SOSIP.v4.2 (PDB entry 6NC3; pink). (B) JR-FL SOSIP.664 (PDB entry 5FYK; green). The RMSD (in Å) are indicated for gp120 and gp41.

We used cryo-EM to generate a high-resolution structure of the AMC009 SOSIP.v4.2 trimer in complex with bNAb PGV04, which targets the CD4-binding site (added as a Fab). We obtained a 3D reconstruction at a resolution of 4.3 Å (PDB entry 6VO3) (Fig. 2; Fig. 3 displays additional information). An overlay showed that AMC009 SOSIP.v4.2 trimers were structurally very similar to BG505 SOSIP.664 (PDB entry 4TVP), with a root mean square deviation (RMSD) of 2.623 Å for gp120 and 1.624 Å for gp41 (37) (Fig. 2). One notable difference was that the longer V1 region of the AMC009 SOSIP.v4.2 trimer adopts a more disordered conformation than its BG505 counterpart. Comparisons with the AMC011 SOSIP.v4.2 (PDB entry 6NC3) (38) and JRFL SOSIP.664 (PDB entry 5FYK) (39) structures yielded similar outcomes (RMSD for AMC009 versus AMC011 of 1.502 Å in gp120 and 1.640 Å in gp41; RMSD for AMC009 versus JRFL of 2.485 Å in gp120 and 2.252 Å in gp41) (Fig. 4A and B, respectively). The AMC009 SOSIP trimer has a longer V5 than the AMC011 and JRFL SOSIP trimers.

### The AMC009 and AMC011 SOSIP trimers have an apparent intact glycan shield dominated by oligomannose glycans.

No conserved potential *N*-glycosylation sites (PNGS), defined as ones present in >50% of HIV-1 group M viruses, were missing from the AMC009 and AMC011 trimer sequences (Table 3) (40–43). In contrast, the AMC008, BG505, and B41 trimers lack conserved PNGS at position 234, positions 130, 241 and 289, or positions 130 and 289, respectively. For the BG505 and B41 SOSIP trimers, the resulting holes in the glycan shields dominate the autologous NAb responses to these trimers (44, 45).

**TABLE 3:** Potential N-linked glycosylation sites^*a*^

SOSIP construct/PNGS	AMC009	AMC011	AMC008	BG505	B41	SF162P3	REJO	WITO
88	N	N	N	N	N	N	N	N
130	N	N	N					
156	N	N	N	N	N	N	N	N
160	N	N	N	N	N	N	N	N
197	N	N	N	N	N	N	N	N
234	N	N		N	N	N	N	
241	N	N	N		N	N	N	N
262	N	N	N	N	N	N	N	N
276	N	N	N	N	N	N	N	N
289	N	N	N				N	
295	N	N	N	N	N	N	N	N
301	N	N	N	N	N	N	N	N
332	N	N	N	N	N	N		N
339	N	N	N	N	N	N	N	N
356	N	N	N	N	N	N	N	N
386	N	N	N	N	N	N		N
392	N	N	N	N	N	N	N	N
448	N	N	N	N	N	N	N	N
611	N	N	N	N	N	N	N	N
616	N	N	N		N	N	N	N
625	N	N	N	N	N	N	N	N
637	N	N	N	N	N	N	N	N
No. of conserved PNGS	21	21	20	18	19	19	18	18
Conserved glycan(s) missing			N234	N130	N130	N130	N130	N130
				N241	N289	N289	N332	N234
				N289			N386	N289
				N625				
Total glycan hole area (Å^2^)	172	33	1153	2377	1649	1747		

a Presence of conserved potential *N*-glycosylation sites (PNGS) in various SOSIP trimers. The numbered sites are conserved in >50% of HIV-1 group M sequences (40–43). An N indicates the presence of a PNGS, a gray background an NxT motif, and a white background an NxS motif. The numbers of conserved PNGS and the predicted accessible protein surface area, in Å (calculated with the Los Alamos Glycan Shield Mapping tool [56]), were used to plot Fig. 5A.

The above-described primary sequence analysis does not factor in 3D protein structure. The Los Alamos Glycan Shield Mapping (LAGSM) tool calculates the surface area, in square angstrom, that is not covered by conserved glycans, taking into account the conserved PNGS present, the 3D structure of the Env protein, and the shielding effect of neighboring PNGS (46). We refer to the predicted accessible protein surface area in square angstrom. The CD4-binding site, the gp120-gp41 interface, and the fusion peptide are never either glycosylated or shielded by nearby PNGS and, therefore, were excluded from the analysis. The LAGSM tool confirmed that both AMC009 and AMC011 SOSIP trimers possess an apparent intact glycan shield. Thus, the predicted accessible protein surface areas of the AMC009 and AMC011 trimers are 172 Å^2^ and 33 Å^2^, respectively, compared to 2,377 Å^2^, 1,649 Å^2^, and 1,153 Å^2^ for their BG505, B41, and AMC008 counterparts (Fig. 5A).

**FIG 5:**
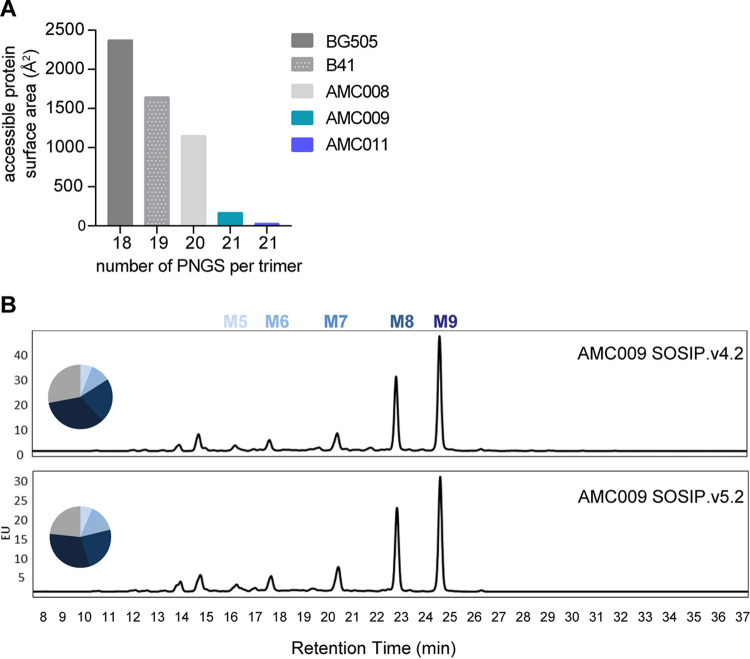
Analysis of the glycan shield of the AMC009 SOSIP.v4.2 and SOSIP.v5.2 trimers. (A) The predicted accessible protein surface area, as defined by the Los Alamos Glycan Shield Mapping tool, is plotted against the number of conserved (i.e., present in >50% of group M HIV-1 isolates) potential *N*-glycosylation sites (PNGS) for the AMC009, AMC011, AMC0008, B41, and BG505 SOSIP trimers (41, 42, 46, 56). The individual PNGS motifs can be found in Table 3. (B) N-linked glycans were assessed by hydrophilic interaction chromatography-ultraperformance liquid chromatography. Peaks representing oligomannose glycans (Man_5-9_GlcNAc_2_) are indicated. The pie charts represent the total abundance.

To acquire information on the glycan composition of various AMC009 and AMC011 SOSIP trimers, we used hydrophilic interaction chromatography-ultraperformance liquid chromatography (HILIC-UPLC). Underprocessed, oligomannose-type glycans dominated the glycan composition of both AMC009 (72% and 77% for SOSIP.v4.2 and SOSIP.v5.2) and AMC011 (72% and 78% for SOSIP.v4.2 and SOSIP.v5.2) trimers (Fig. 5B and Table 2). These findings are qualitatively similar to those for other native-like SOSIP trimers, although the oligomannose contents of the AMC009 and AMC011 trimers are at the high end of the observed range (29). Thus, for comparison, 63% of the glycans on BG505 SOSIP.664 are of the oligomannose type (47). The high percentage of oligomannose glycans may reflect the high density of glycans on the AMC009 and AMC011 trimers and the consequently reduced access of some glycans to endoplasmic reticulum and Golgi glycan processing enzymes (Fig. 5A and Table 2) (48).

**FIG 6:**
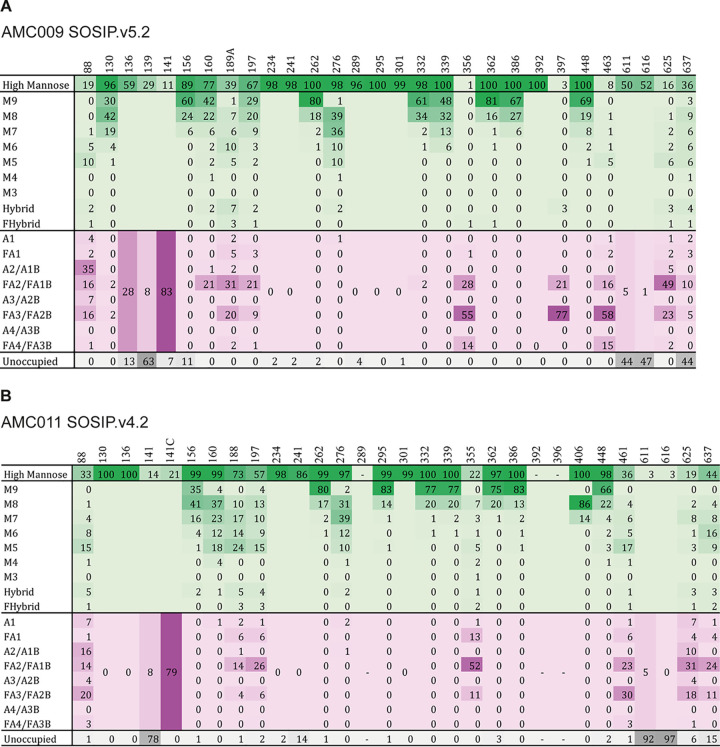
Site-specific glycan analysis. Quantification of site-specific glycan occupancy and composition derived from liquid chromatography-mass spectrometry experiments on all 29 potential *N*-glycosylation sites (PNGS). The tables show the glycoforms found at each PNGS. Compositions corresponding to oligomannose/hybrid-type glycans are colored green, and fully processed complex type glycans are colored magenta. PNGS with no attached glycan are colored gray. Oligomannose-type glycans are categorized according to the number of mannose residues present, hybrid-type glycans are categorized according to the presence/absence of fucose, and complex-type glycans are categorized according to the number of processed antennas and the presence/absence of fucose. (A) AMC009 SOSIP.v5.2 trimer. (B) AMC011 SOSIP.v5.2 trimer (D7324-tagged). Sites N289, N392, and N396 could not be resolved.

Site-specific glycan composition information on the AMC009 SOSIP.v5.2 and AMC011 SOSIP.v5.2 trimers was obtained using liquid chromatography-mass spectrometry (LC-MS) (Fig. 6A and B, respectively). Overall, these patterns are similar to observations made with BG505 SOSIP.664 (47, 49). On the AMC011 SOSIP.v5.2 trimer, nearly all N301 glycans were of the oligomannose type (99% oligomannose), which is in contrast to a high abundance of complex glycans at N301 for AMC011 SOSIP.664 (50). The difference can be explained by a more stable conformation of AMC011 SOSIP.v5.2, resulting in a more restricted access of the glycan trimming and processing enzymes.

In summary, the stabilized AMC009 and AMC011 SOSIP trimers possess an intact glycan shield, and the majority of PNGS are occupied by oligomannose-type glycans.

### AMC009 and AMC011 SOSIP trimers induce autologous NAbs weakly.

We conducted an immunization study to test two hypotheses. First, SOSIP trimers derived from elite neutralizers might give rise to bNAbs. Second, Env trimers without immunodominant glycan holes might induce a heterologous NAb response instead of a strain-specific autologous response.

**FIG 7:**
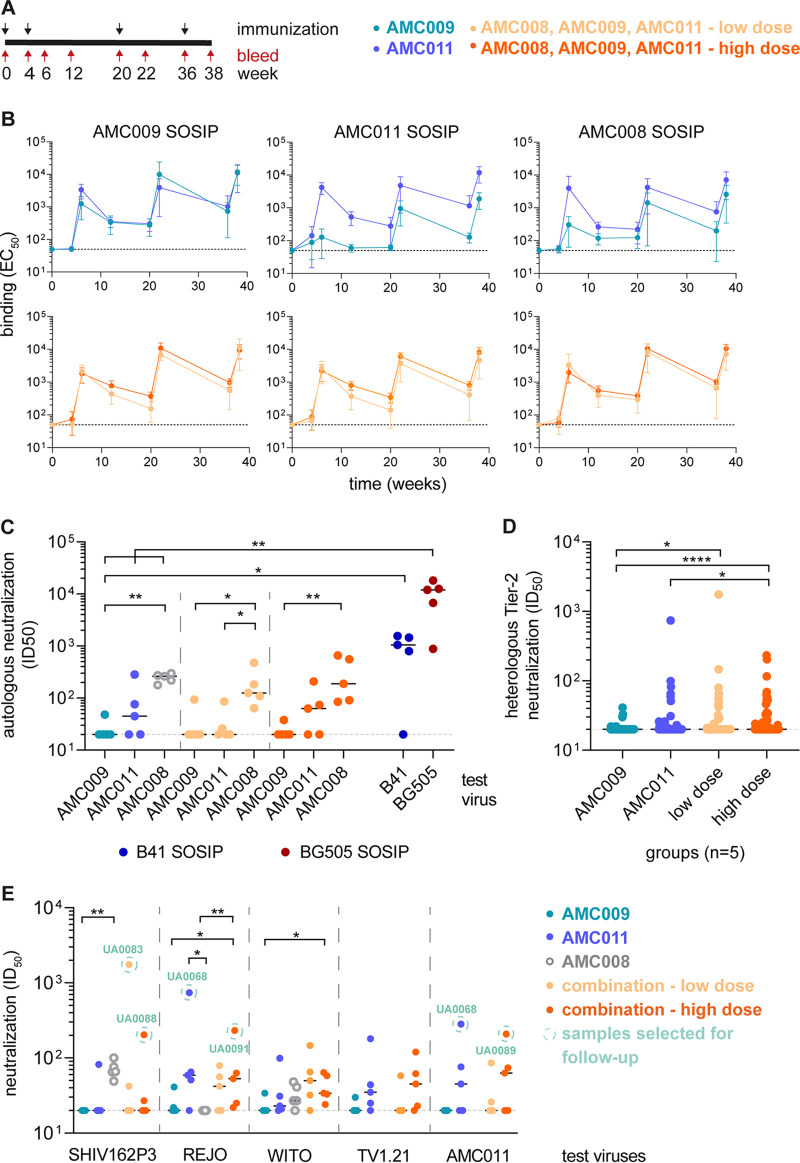
Immunogenicity of AMC009 and AMC011 SOSIP trimers in rabbits. (A) Immunization schedule. Four groups of rabbits (*n* = 5) were immunized at weeks 0, 4, 20, and 36 (black arrows) with either SOSIP.v4.2 (first three doses) or SOSIP.v5.2 (final dose). Sera were obtained at weeks 0, 4, 6, 12, 20, 22, 36, and 38 (red arrows). In the monovalent immunogen groups, the animals received 22 μg of trimer per dose. Two trivalent combinations were also tested: in the low-dose group the animals received 7.3 μg of each trimer, 22 μg in total; in the high-dose group 22 μg of each trimer was given, 66 μg in total. The AMC008 SOSIP.v4.2 trimer was not used in this experiment, but historical neutralization data from the week 22 time point are plotted for comparison for panels C and E (19). (B) Antibody-binding titers (50% effective concentration [EC_50_]) against His-tagged AMC009 SOSIP.v5.2, AMC011 SOSIP.v5.2, and AMC008 SOSIP.v4.2 trimers were measured by Ni-NTA ELISA. The mean EC_50_ values are plotted with the standard deviations shown. (C) Autologous neutralization titers (ID_50_) in week 38 sera. One virus was tested for the monovalent immunogen groups, all three for the trivalent groups. The AMC009 and AMC011 viruses are tier 2, and AMC008 is tier 1B. Historical week 22 ID_50_ values of BG505 SOSIP.v5.2 and B41 SOSIP.v4.1 immunized animals are plotted for comparison with the monovalent groups (31). (D) Heterologous neutralization titers (ID_50_) in week 38 sera against 16 tier 2 viruses, including nine viruses of the global virus panel (82). (E) Neutralization titers (ID_50_) against four heterologous tier 2 viruses and the parental AMC011 virus at week 38. Circled are the serum-virus combinations used for the EM-based polyclonal epitope mapping analysis in Fig. 9B. The dashed line indicates the lower assay cutoff ID_50_ value of 20, and the horizontal black line shows the median titer per group. Indicated are the statistical significances. Individual ID_50_ values are reported in Table S1 in the supplemental material.

In an initial study, rabbits were immunized four times with 22 μg of either AMC009 SOSIP or AMC011 SOSIP trimer (Fig. 7A; see also Materials and Methods). The first three immunizations were performed using SOSIP.v4.2 trimers, while SOSIP.v5.2 trimers were used for the final one, as they had become available. Binding antibody titers against the AMC009 and AMC011 trimers, and the heterologous AMC008 trimer, were quantified longitudinally. Both immunogen trimers induced autologous binding antibody titers that waned after each dose and were then reboosted; the peak titer was at week 38, after the fourth immunization (Fig. 7B). Antibodies elicited by the AMC009 trimers were markedly less reactive with the heterologous AMC011 and AMC008 trimers than with the autologous trimer (fold difference of 5 and 3, respectively, in 50% effective concentration compared to those of the autologous trimer). In contrast, sera from the AMC011 trimer-immunized animals bound comparably to all three trimers (Fig. 7B).

The NAb responses against autologous and heterologous viruses were analyzed at week 22 and week 38 (see Table S1 in the supplemental material), i.e., 2 weeks after the third and fourth immunizations. The breadth and potency of the antibody response increased from week 22 to week 38. The following analyses are based on week 38 data (Fig. 7C to E). NAb responses against murine leukemia virus (MLV), serving as a negative control, were undetectable, except for animal UA0069, which had an ID_50_ of 22 (Table S1). Both the AMC009 and AMC011 SOSIP trimers induced NAbs against tier 1 viruses SF162, BaL, and ZM109F (Table S1).

The AMC011 SOSIP trimer induced autologous NAbs weakly (median ID_50_ of 45), whereas animals immunized with the AMC009 trimer did not neutralize the autologous virus (median ID_50_ of <20) (Fig. 7C). For comparative reasons, historical autologous neutralization data based on sera from AMC008 SOSIP.4.2 trimer-immunized rabbits (week 22; 3 immunizations; formulated in ISCOMATRIX; *n* = 5) were included in Fig. 7C (19). The autologous NAb titers for the AMC008 group were significantly higher than those for the AMC009 group (median ID_50_ values of 262 versus 20; *P* < 0.01) but not the AMC011 group.

B41 SOSIP.v4.1 and BG505 SOSIP.v5.2 trimers are known to induce a strong autologous NAb response due to immunodominant holes in the glycan shield (22). The autologous NAb response induced by AMC009 was significantly lower than the NAb response of B41 SOSIP.v4.1-immunized animals (*P* < 0.05; historical week 22 data [31]). Rabbits of the three monovalent groups had a significantly lower autologous NAb response than BG505 SOSIP.v5.2-immunized animals (*P* < 0.01; historical week 22 data) (Fig. 7C).

The AMC009 trimer elicited very low or nonexistent heterologous tier 2 NAbs, but these responses were sometimes seen in the AMC011 group (Fig. 7D and E and Table S1). Four sera from the AMC011 group neutralized the tier 2 subtype B virus REJO4541.67 (median ID_50_ of 59). Serum from the AMC011 SOSIP-immunized animal UA0069 cross-neutralized the subtype B tier 2 viruses REJO, SHIV162P3, 92BR020, and WITO4160.33 at low levels (ID_50_ of 51, 82, 81, and 99, respectively) (Table S1). TV1.21, a subtype C virus, was neutralized by animals UA0069 and UA0070 (ID_50_ of 180 and 44) (Table S1). Overall, there was no significant difference between the median heterologous neutralization titers (ID_50_) for the AMC011 and AMC009 groups (Fig. 7D). We did note that more tier 2 viruses were hit, including TV1.21 and the global virus panel, with an ID_50_ of >20, by the AMC011 sera than AMC009 sera (19 and 10, respectively). When the cutoff for this analysis was raised to an ID_50_ value of >40, i.e., 2-fold over background, the corresponding number of hits for AMC011 and AMC009 groups was 7 and 1, respectively (individual ID_50_ values are reported in Table S1).

We conclude that the AMC008, AMC009, and AMC011 SOSIP trimers induce substantially weaker autologous NAb titers than other SOSIP trimers, such as those derived from the B41 and BG505 isolates (31). The AMC011 trimer was the more immunogenic of the two, and, unlike AMC009, it was also able to elicit sporadic low-level NAbs against heterologous tier 2 viruses.

### Combining AMC009, AMC011, and AMC008 trimers induces tier 2 NAbs in rabbits.

We previously found that trivalent and tetravalent combinations of SOSIP trimers from subtypes A, B, and C did not elicit heterologous NAbs effectively, because autologous NAbs against the glycan holes present on the individual trimers seem to dominate over cross-reactive responses (30, 31). We hypothesized that antigenically diverse trimers (i.e., from subtypes A, B, and C) are unable to drive cross-reactive NAb responses due to the lack of shared epitopes. Accordingly, here we have tested whether a trivalent combination of antigenically more similar SOSIP trimers might be more efficient at inducing cross-reactive NAb responses. We selected three SOSIP trimers, AMC008, AMC009, and AMC011, which were all based on subtype B viruses from participants of the ACS who were classified as elite or broad neutralizers and share a sequence identity of 85 to 87% at the amino acid level (19, 33, 51–53). Compared with that of the AMC009 and AMC011 SOSIP trimers, the glycan shield of the AMC008 SOSIP trimer is somewhat less dense, as more surface area is accessible on the trimer (Fig. 5A) because of the absence of the conserved PNGS at position 234 (Table 3). Nevertheless, the exposed surface area was substantially smaller than that of prototypic trimers with holes, such as BG505 SOSIP (19, 44). In addition, animals of the AMC008 SOSIP group sporadically neutralized heterologous subtype B tier 2 viruses (median ID_50_ of 66 for SHIV162P3 and 27 for WITO) (Fig. 7E) (19).

To test the effect of the amount of each SOSIP trimer in the combination group, the individual components were administered at a low concentration, with the overall amount the same as that of the monovalent groups. The animals of the high-dose group received each SOSIP trimer in the same concentration as the monovalent groups (Fig. 7A; see also Materials and Methods).

The animals in both trivalent combination groups generated strong binding Ab responses that followed the same saw-tooth pattern as that for the monovalent immunizations (Fig. 7B). The binding titers against the AMC011 trimer were slightly lower in the low-dose group than in the high-dose group (3-fold difference at weeks 12, 20, 36, and 38). No difference between the two combination groups has been observed in binding to AMC009 and AMC008 SOSIP.

The binding titers to AMC009 SOSIP were similar for both combination groups compared to the monovalent titers. The same comparison was done for AMC008 SOSIP, showing a slight enhancement in the combination groups (∼3-fold for low dose and ∼5-fold for high dose, on average, for all time points) (Fig. 7B). For the AMC011 SOSIP, a 5-fold higher binding titer was observed for the high-dose group and an ∼2-fold lower titer for the low-dose group (on average, for all time points) than for the monovalent group.

The neutralization responses of the week 38 sera from both combination groups were assessed for autologous and heterologous viruses. None of the sera neutralized MLV (Table S1). Week 38 sera from all 10 rabbits neutralized the subtype B tier 1 viruses SF162, BaL, and ZM109F (Table S1).

The autologous NAb responses against the AMC009 and AMC011 viruses were similar in frequency and magnitude to those seen in the corresponding monovalent trimer groups (AMC009, median ID_50_ of 20 for low- and high-dose groups; AMC011, median ID_50_ of 20 for low dose and 63 for high dose) (Fig. 7C). All 10 animals in the combination groups developed autologous NAbs against the tier 1B AMC008 virus (median ID_50_ of 125 for low dose and 188 for high dose), which is comparable to what was described at the week 22 time point for 5 rabbits immunized with monovalent AMC008 trimers (median ID_50_ of 262) (19) (Fig. 7C).

The heterologous tier 2 subtype B viruses WITO (median ID_50_, 106 for low dose and 61 for high dose) and REJO (median ID_50_, 42 for low dose and 53 for high dose) were weakly but consistently neutralized by sera from the combination groups (Fig. 7E). Other heterologous tier 2 viruses, SHIV162P3 and TV1.21, were sporadically neutralized in the low- and high-dose groups (SHIV162P3, median ID_50_ of 20 for both groups; TV1.21, median ID_50_ of 20 and 45 for low and high dose, respectively). In the low-dose group, 2/5 animals neutralized SHIV162P3 (ID_50_ of 42 and 1,749) and 1/5 animals neutralized TV1.21 (ID_50_ of 58). One animal of the high-dose group neutralized SHIV162P3 (ID_50_ of 204), and 3/5 animals neutralized TV1.21 (ID_50_ of 45, 120, and 62) (Fig. 7E and Table S1). In addition, some animals of both combination groups neutralized BG505 T332N, CH119.10, 25710-2.43, TRO.11, and Ce1176_A3 weakly (Table S1). The neutralization titers against REJO in both the low- and high-dose combination groups were similar to those seen in the monovalent AMC011 trimer group (median ID_50_ of 42 and 53, respectively, versus 59), whereas neutralization titers in the AMC009 group and in the historical AMC008 group were low (median ID_50_ of 21 and 20, respectively; *P* < 0.05 for the comparison of AMC009 versus high dose and *P* < 0.01 for AMC008 versus high dose) (Fig. 7E). Neutralization of WITO differed only in comparison to the AMC009 group (*P* < 0.05 for high dose versus AMC009). In contrast, no statistical difference was observed between the monovalent groups and both combination groups in SHIV162P3 neutralization (Fig. 7E).

In comparisons of the tier 2 NAb response of both combination groups with the AMC009 group, differences were observed (*P* < 0.05 for comparison of the low dose versus AMC009 group and *P* < 0.0001 for the comparison of the high dose versus the AMC009 group). In addition, a statistical difference between the high-dose group and the AMC011 group (*P* < 0.05) was apparent (Fig. 7D). Overall, more heterologous tier 2 viruses were neutralized in the combination groups than in the two monovalent groups (2-fold difference for ID_50_ of >20 and 2.5-fold difference for ID_50_ of >40).

**FIG 8:**
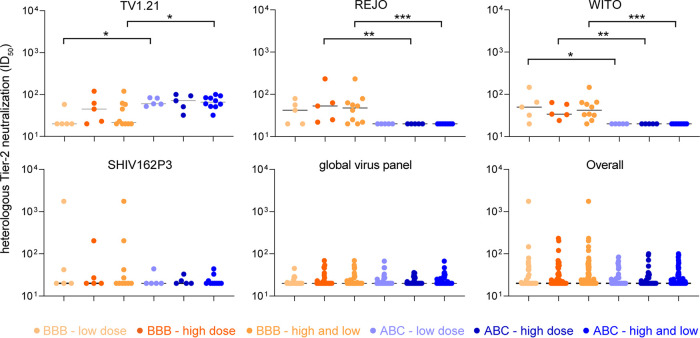
Comparison of heterologous tier 2 neutralization (ID_50_) induced by the trivalent subtype B combination (AMC009, AMC0011, and AMC008 SOSIP.v5.2) versus historical data of a trivalent subtype A, B, and C combination, consisting of BG505, AMC008, and B41 SOSIP.v4 variants (week 38 versus week 22; ISCOMATRIX; *n* = 5) (31). Test viruses are indicated, and data for all test viruses were combined in the last panel (“overall”). ID_50_ values of the low- and high-dose groups are plotted individually as well as combined.

Comparing the heterologous tier 2 NAb response of the trivalent subtype B combination (BBB) with a trivalent subtype A, B, and C combination (ABC; BG505, AMC008, and B41 SOSIP.v4 variants; week 38 versus week 22; ISCOMATRIX; *n* = 5) (31) revealed that the BBB combination induced lower neutralization titers against TV1.21 (*P* < 0.05 for BBB low versus ABC low and *P* < 0.001 for BBB versus ABC), higher titers against REJO (*P* < 0.01 for BBB high versus ABC high and *P* < 0.001 for BBB versus ABC), and higher titers against WITO (*P* < 0.05 for BBB low versus ABC low, *P* < 0.01 for BBB high versus ABC high, and *P* < 0.001 for BBB versus ABC) (Fig. 8). No significant difference was observed in neutralization titers against SHIV162P3, the global virus panel, and the overall NAb response against all 13 heterologous tier 2 viruses.

To summarize, the BBB combination seems to be more effective at inducing neutralization titers against subtype B viruses than the ABC combination.

### Autologous and heterologous tier 2 antibodies cross-react with trimers from multiple isolates.

To characterize the heterologous tier 2 NAbs, we selected six serum-virus combinations based on five sera with the highest heterologous neutralization titers (ID_50_ titer of >100) (indicated in Fig. 7E). Four sera were from rabbits immunized with the trivalent combination of AMC008, AMC009, and AMC011 trimers (UA0083, UA0088, UA0089, and UA0091), while a fifth rabbit received only the AMC011 trimer (UA0068).

**FIG 9:**
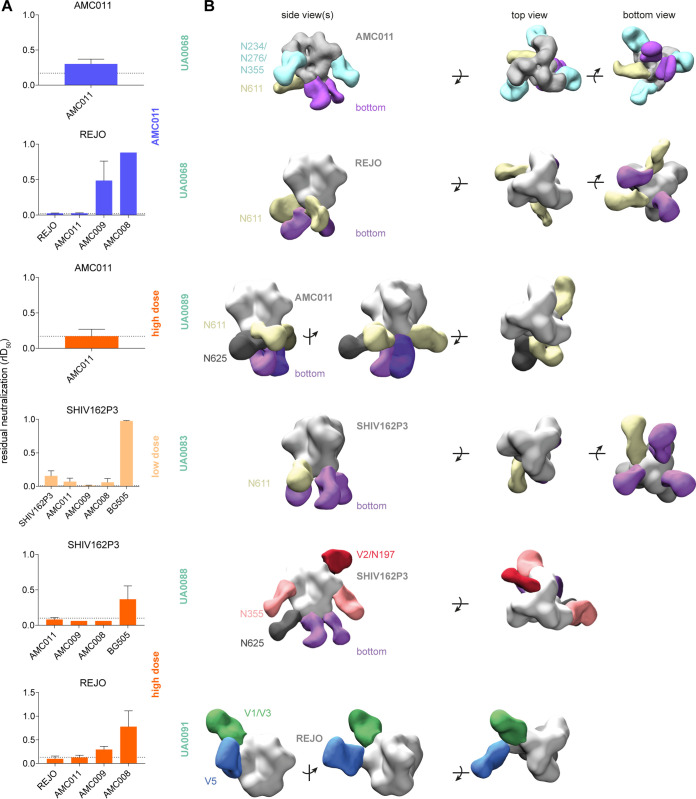
Characterization of the antibody specificities of immunized rabbits at week 38. (A) Neutralization depletion data (see Materials and Methods for further information). SOSIP trimers corresponding to the immunogens (AMC009, AMC011, and AMC008 SOSIP) and SOSIP proteins derived from heterologous tier 2 viruses (REJO4160.33, SHIV162P3, and BG505) were used as depleting reagents and are listed on the *x* axis. Indicated are the test viruses (graph heading), animal IDs (boldface green), and the immunization group. The ID_50_ titer of neutralization in the absence of a depleting reagent was set at 1. The neutralization in the presence of depleting reagents was normalized accordingly and presented as relative ID_50_ (*r*ID_50_). Full depletion is indicated by the dashed line and varies per serum. For UA0068 against AMC011, the *r*ID_50_ equaling full depletion was calculated individually. (B) EM-based polyclonal epitope mapping. Fabs of antibodies purified from rabbit sera were complexed with a SOSIP trimer, corresponding to an autologous or heterologous virus (see Fig. 7E, circled in green). The binding was assessed via NS-EM (54). The targeted epitopes are indicated.

Various SOSIP trimers were used in neutralization-depletion assays to assess whether the serum NAbs were trimer reactive (Fig. 9A) (19). To do so, the sera were incubated with SOSIP trimers as depleting reagents. After incubation, the virus was added to the mixture (see Materials and Methods for more information) and the ID_50_ relative to the ID_50_ without depleting reagent (*r*ID_50_) calculated. Full depletion of neutralization is indicated by the dashed line and varies per serum. The test panel included the autologous immunogen trimers (AMC008, AMC009, and AMC011) and trimers corresponding to the heterologous viruses that were neutralized (REJO and SHIV162P3). In some cases, the B41 and BG505 SOSIP trimers were also tested.

Neutralization of REJO by the UA0068 and UA0091 sera was completely depleted, or nearly so, by the REJO and AMC011 SOSIP trimers (Fig. 9A). The AMC009 trimer nearly fully depleted REJO neutralization for UA0091 but only partially for UA0068, whereas the AMC008 trimer barely depleted neutralization.

In contrast, the NAbs against SHIV162P3 in sera from animals UA0083 and UA0088 were almost completely depleted by all three immunogen trimers (Fig. 9A). This observation is concordant with the finding that both sera cross-neutralized all three autologous viruses (AMC008, AMC009, and AMC011). Hence, it is possible that all three trimer immunogens contributed to the heterologous neutralization of SHIV162P3. In contrast, BG505 SOSIP was unable to completely deplete the SHIV162P3 NAb activity from these sera.

### Polyclonal antibodies target diverse epitopes on autologous and heterologous trimers.

To further investigate autologous and heterologous antibody specificities, we performed EM-based polyclonal epitope mapping (EMPEM) (54), using selected sera from which the neutralization activity could be efficiently depleted by a SOSIP trimer (Fig. 9A). EMPEM allows the visualization of the highest affinity and most abundant Abs within a polyclonal response (54). Serum IgG was purified and digested with papain to obtain Fabs, which were then incubated with a trimer to form complexes for imaging by NS-EM. As serum volumes were limited, we assessed antibody binding only to heterologous trimers, with the exception of serum UA0068, for which we tested the autologous AMC011 trimer and the heterologous REJO trimer.

The serum antibodies induced in rabbit UA0068 by the AMC011 trimer targeted three distinct epitopes on the autologous AMC011 trimer. One of them is the base of the trimer (Fig. 9B, purple), eliciting a dominant but nonneutralizing antibody response. An antibody response against the base is consistent with previous findings and with the presence of a large glycan hole caused by the absence of the membrane. The other two targeted epitopes are at or near the N611 glycan (Fig. 9B, yellow) and a region between the glycans at N234, N276, and N355 (Fig. 9B, cyan). The first two epitopes were also targeted on the heterologous REJO trimer (Fig. 9B, purple and yellow, respectively), but no Fab binding to the region between N234, N276, and N355 glycans was visible. Hence, antibodies to this site appear to be an autologous AMC011-specific response.

A similar cross-reactivity pattern was seen with serum UA0083 and the SHIV162P3 SOSIP trimer, as both the base (Fig. 9B, purple) and the N611 glycan site (Fig. 9B, yellow) were targeted. However, the antibodies approached the N611 glycan site from a different angle than how their UA0068 counterparts bound to a similar site on the REJO trimer.

Four dominant antibody specificities were seen in the complex between UA0089 Fabs and the autologous AMC011 trimer. One target was the N611 glycan site (Fig. 9B, yellow), while a second was at or near the N625 glycan (Fig. 9B, black). Two different varieties of base binding Fabs were also seen (Fig. 9B, purple and lilac). The Fab targeting the N611 glycan site did so via an approach angle that differed from both of those seen with the UA0068 and UA0083 sera noted above.

There were clear similarities in the Fab binding patterns described above. However, the antibody specificities detected in the other three sera were quite different. In UA0091, two epitopes in the V1/V3 and V5 domains were targeted on the REJO trimer (Fig. 9B, green and blue), and no cross-binding Fabs against the base were detected. The Fabs from serum UA0088 targeted four different epitopes on the SHIV162P3 SOSIP trimer. One epitope was at or near the V2 apex, potentially involving the N197 glycan (Fig. 9B, red), and a second was located around the N355 glycan (Fig. 9B, pink). As observed in animal UA0089, the N625 site was also targeted (Fig. 9B, black), as was the trimer base (Fig. 9B, purple).

In summary, four different cross-reactive epitopes on the REJO trimer were targeted by the two sera that neutralize the REJO virus, while five different epitopes on the SHIV162P3 trimer were targeted by the two sera that neutralized this virus. The trimer base was targeted in four of five animals, and in three of them these nonneutralizing antibodies were cross-reactive with heterologous trimers. The area around the N611 glycan was targeted in three of five animals, in two cases by cross-reactive antibodies.

**FIG 10:**
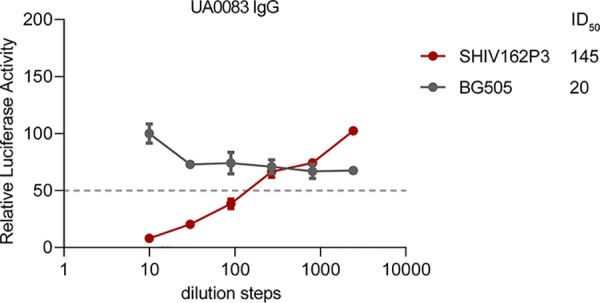
Neutralization of purified serum IgG from animal UA0083 against SHIV162P3 and BG505. The neutralization titers (ID_50_) are indicated. For more information, see Materials and Methods.

The purified serum IgG of animal UA0083 that was used for the EMPEM analysis was also tested in a neutralization assay (see Materials and Methods). SHIV162P3 was neutralized with an ID_50_ of 145 (Fig. 10), whereas the BG505 virus was not neutralized.

### PNGS in gp41 are underoccupied on AMC009 and AMC011 SOSIP trimers.

Antibodies recognizing the N611 glycan have been described previously (85), as have antibodies induced by BG505 SOSIP trimers that can only neutralize BG505 virus mutants lacking the N611 glycan (R. Derking and R. W. Sanders, submitted for publication). To better understand the N611 glycan response seen here in sera UA0068, UA0083, and UA0089, we determined whether the N611 PNGS and others were fully occupied on the AMC009 and AMC011 trimer immunogens using site-specific glycan analysis.

**FIG 11:**
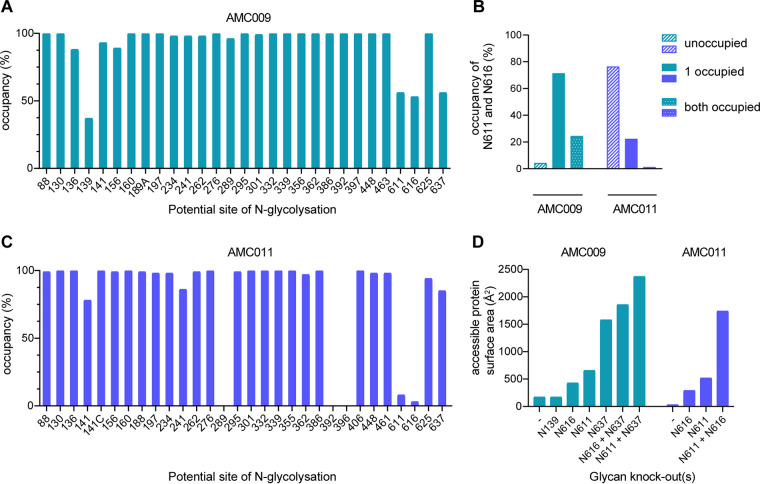
Analysis of glycan occupancy of the AMC009 and AMC011 trimers. Site-specific glycan occupancy of the AMC009 SOSIP.v5.2 and AMC011 SOSIP.v5.2-D7324 trimers. The proportion of glycopeptides at each potential *N*-glycosylation site were compared to those lacking a glycan to determine the occupancy at each site using liquid chromatography-mass spectrometry. Individual values can be found in Fig. 6. (A) Glycan occupancy of the AMC009 SOSIP trimer. (B) Occupancy of AMC009 (turquoise) and AMC011 (purple) of N611 and N616. Bars indicate the absence of glycans at both positions, the presence of a glycan at one position, and the occupancy of both positions. (C) Glycan occupancy of the AMC011 SOSIP trimer. The occupancy at positions 289, 392, and 396 could not be determined. (D) Potential *N*-glycosylation sites were knocked out in the sequences of AMC009 and AMC011, which have been shown to be more than 40% unoccupied in site-specific analysis (A and B). The predicted glycan unshielded area was calculated for each glycan KO using the Los Alamos Glycan Shield Mapping tool (46).

For AMC009 SOSIP.v5.2 trimers, the majority of sites were fully occupied, including ones, such as N332 and N160, that are critical for bNAb recognition (Fig. 11A). The occupancy on the gp120 subunits was lower at sites where PNGS are close together, notably in the V1/V2 region, which is consistent with a previous report on closely spaced PNGS (55). Occupancy was <90% at sites N136, N139, and N156 (87%, 37%, 89%, respectively), and it was substantially reduced at gp41 sites N611, N616, and N637 (66%, 53%, 66%, respectively) (Fig. 6A). Analysis of individual peptides containing both N611 and N616 showed a nonrandom distribution of occupancy, in that in most cases either site was occupied but not both (Fig. 11B). Peptides containing two glycans were infrequent (24%), while peptides containing no glycan were rare (4%). Thus, the majority of AMC009 SOSIP trimers contain a glycan at either the N611 or the N616 site (72%).

A similar pattern was seen for the AMC011 SOSIP.v5.2 trimers. Most sites were fully occupied, although <90% occupancy was seen for N141 and N241 (22% and 86%, respectively), and greatly reduced occupancy was also found at gp41 sites N611, N616, and N637 (8%, 3%, and 85%, respectively) (Fig. 11C and 6B). The N611 and N616 occupancy is in stark contrast to that of the AMC009 SOSIP trimer (66% and 53%, respectively). Peptides containing both the N611 and N616 glycans were very rare (1%), as were peptides that contain a glycan at either the N611 or N616 site (22%) (Fig. 11B). The majority of AMC011 SOSIP trimers presented no glycans at position N611 or N616 (76%), which is in stark contrast to the AMC009 SOSIP trimer (4%).

To study the impact of underoccupied AMC009 PNGS motifs on the overall glycan shield, we knocked out the NxT/S motifs for N139, N611, N616, and N637 (positions that are occupied in <60% of cases) *in silico* via NxK motifs and then reanalyzed the accessible protein surface area of the modified sequences using the LAGSM tool (56). As expected, the accessible protein surface area increased with each modification, with the N637 knockout having the greatest impact (1,579 Å^2^ compared to 172 Å^2^ for the unmodified trimer) (Fig. 11D). The largest accessible protein surface area was created by simultaneously removing the N611 and N637 glycans (2,373 Å^2^). The N139 knockout had no effect on the total accessible protein surface area, probably because there is a dense glycan shield in that region.

Similarly, we knocked out PNGS motifs in AMC011 that were occupied <60% (N611, N616, and N611+N616) (Fig. 11D). The accessible protein surface area increased with the individual modifications of N611 or N616 (519 Å^2^ and 290 Å^2^, respectively, compared to 33 Å^2^ for the wild-type sequence). N637 was not analyzed, as this site is occupied to an extent of >60% (i.e., 85%). In contrast to AMC009, AMC011 has a higher frequency of peptides that do not contain N611 and N616 (4% for AMC009 versus 76% for AMC011). Removing both N611 and N616 resulted in the largest accessible protein surface area (1,740 Å^2^).

This analysis shows that the least occupied AMC009 and AMC011 trimer variants, as determined *in silico*, have accessible protein surface areas similar to or slightly less than those of BG505 SOSIP trimers (3,117 Å^2^, taking an occupancy of <60% into account [57]). Overall, the combined underoccupancy of N611/N616 and N637 for AMC009 and N611 and N616 for AMC011 might result in a large accessible protein surface area. This could explain why antibodies are induced against this region of the trimer (Fig. 9B).

In addition, the EMPEM analysis of AMC011 SOSIP complexed with Fabs of animal UA0068 revealed an antibody specificity targeting the region of N234, N276, and N355 (Fig. 9B, cyan). All three PNGS are nearly fully occupied (98%, 100%, and 100%) (Fig. 11C and 6B), suggesting an involvement of the glycans in antibody binding or antibody navigation through the glycans.

## DISCUSSION

We report on a novel stable and native-like SOSIP trimer, termed AMC009 SOSIP, derived from an elite neutralizer, and describe its design, structure, biochemical and biophysical properties, antigenicity, and immunogenicity. Furthermore, we generated a more stable version of the AMC011 SOSIP trimer, also derived from an elite neutralizer, by introduction of the C73-C561 disulfide bond (29, 33). Both trimers have all the hallmarks of stable and native-like Env trimers that expose bNAb epitopes while occluding most non-NAb epitopes. A 4.3-Å-resolution structure, resolved by single-particle cryoelectron microscopy (cryo-EM), revealed that the AMC009 SOSIP trimer indeed resembles the native Env trimer. In contrast to the prototypic BG505 and B41 SOSIP trimers, the AMC009 and AMC011 trimers appear to possess a more complete glycan shield.

One underlying reason for selecting the AMC009 and AMC011 sequences was the hypothesis that early *env* sequences from elite neutralizers are more efficient at inducing bNAbs, especially if they possess an intact glycan shield (9, 33, 56). Both AMC009 and AMC011 SOSIP-immunized animals developed poor autologous NAb responses compared to the BG505 and B41 SOSIP-immunized animals, which probably can be explained by the absence of immunodominant glycan holes that usually dominate the induction of strain-specific autologous NAb responses against SOSIP trimers (20, 44, 58). Comparing the AMC009 and AMC011 trimers, we observed that more animals of the AMC011 group than the AMC009 group neutralized the autologous virus, which could be accounted for by difference in glycan occupancy or the accessibility of the epitope that led to the induction of bNAbs in natural infection. We note that our data, in combination with published data, suggest that the autologous neutralization titers that are induced by SOSIP trimer vaccination correlate with the accessible protein surface area. However, more SOSIP trimers need to be evaluated to draw firm conclusions.

Wagh et al. associated an intact glycan shield of a transmitter and founder virus with a faster development of bNAb responses (56). Our results with AMC011 SOSIP might be consistent with this hypothesis, but the results obtained with the AMC009 SOSIP trimer are not. While the AMC011 trimer induced somewhat better heterologous NAb responses than BG505 and B41 trimers, for example, neither AMC009 SOSIP nor AMC011 SOSIP induced bNAb responses. Taking the intact glycan shield and the fact that AMC009 SOSIP is based on early viruses after seroconversion into account, we expected that the AMC009 trimer would induce NAbs, at least in a range similar to that of AMC011 SOSIP. Many reasons might underlie the difference with the human individuals that were infected with the AMC009 and AMC011 viruses, but the most prominent difference might be that in these individuals the continuous virus evolution might have contributed to shaping the bNAb response. Immunization with one immunogen derived from an elite neutralizer does not recapitulate this process. Multiple immunogens derived from the evolving viral quasispecies might mimic this process, and we are currently generating multiple longitudinal SOSIP trimers from the AMC009 and AMC011 individuals to explore this further.

The hypothesis behind an immunization with a trivalent combination of SOSIP trimers is to focus the immune response of shared (i.e., broadly reactive) epitopes. As suggested by Klasse et al. and Torrents de la Peña et al., the combination immunogen should not be genetically too distinct, which holds true for the AMC009, AMC011, and AMC008 SOSIP trimers (19, 30, 31, 33). The most striking difference between monovalent and trivalent combination groups was observed in the NAb response against heterologous tier 2 viruses, especially compared to monovalent AMC009 SOSIP-immunized animals. The NAb response differed per virus as well as between the three monovalent groups and both combination groups. The high-dose combination group was the most efficient at inducing tier 2 NAb responses.

Compared to a trivalent ABC combination, we observed the trend that animals immunized with a trivalent subtype B combination neutralized more viruses within subtype B and neutralized them at a higher magnitude. One explanation is that shared epitopes, present on subtype B viruses, are being targeted and independent autologous NAb responses are not elicited, as seen with the ABC combination (31). This is in line with a previous observation that subtype B-infected individuals neutralize subtype B viruses better than viruses from other subtypes (59).

According to the EMPEM analysis, two antibody specificities dominated the antibody response. The first involves responses against the base of the trimer. Some base binders, but not all, recognized the heterologous SOSIPs, suggesting a certain level of cross-reactivity. The immunodominance of the trimer base was previously shown and is an inevitable result of immunization with soluble SOSIP trimers (23, 24, 54). Future trimer immunogen improvement programs might need to take this information into account and include strategies to limit the immunogenicity of the trimer base.

The second dominant response involved specificities directed at N611, which cross-bound the autologous and heterologous SOSIP trimers. The underoccupancy of the N611 and N616 PNGS on both AMC009 and AMC011 SOSIP trimers is consistent with this region being immunogenic. Cao at el. described an underoccupancy of SOSIP trimers at N611 as well as a difference in the glycan composition and occupancy between viral strains (49, 60). As we do not know the occupancy of AMC009, AMC011, and the other neutralized viruses, we cannot rule out that they are underoccupied at N611.

The LAGSM tool provides a first idea on the density of the glycan shield of *env* sequences. However, our analyses reveal how this tool might be improved by taking into account whether a PNGS is, in fact, occupied by a glycan.

Several recent immunization studies with Env trimers have yielded heterologous tier 2 NAb responses, although they remain sporadic and/or weak (61, 62). Future goals are now to further strengthen and broaden these NAb responses, leading to a neutralization of more tier 2 viruses from different subtypes, and to have a more consistent NAb response in all immunized animals. These goals might be aided by displaying the immunogens of the trivalent combination together on nanoparticles, focusing the immune response on a shared epitope(s) on all three proteins and hiding the base (23, 24, 63). Another possibility to guide the immune system toward the production of broadly neutralizing antibodies is to immunize, in a sequential fashion, using natural infection in an elite neutralizer as a blueprint (32). A third strategy that we favor involves priming with structure-based germ line-targeting immunogens, “shaping” with affinity intermediates, and “polishing” with trimers that possess an intact and dense glycan shield without immunodominant holes (22, 61, 64, 65). Both AMC009 and AMC011 SOSIP trimers might be excellent immunogens for boosting antibody responses that were primed with a germ line-targeting immunogen and helping the antibodies to mature and to recognize glycans.

In conclusion, stabilized Env trimer immunogens derived from subtype B HIV-1-infected elite neutralizers from the Amsterdam cohort induced heterologous tier 2 NAb responses within subtype B, some of those occurring after three immunizations. Although these responses were sporadic, some animals neutralized heterologous primary virus isolates at high titer. Further analysis provided insights into combination immunization and the impact of the HIV-1 Env glycan shield on immunogenicity. Even though AMC009 SOSIP did not induce a NAb response on its own, it might contribute to the NAb response seen in animals immunized with a trivalent combination of SOSIP trimers. A further understanding of the differences in glycosylation between SOSIPs and viruses, as well as an enhanced understanding of the antibody-virus coevolution in AMC009 and the targeted epitope(s), will help to further improve the immunogenicity of the AMC009 SOSIP trimer. Overall, AMC009 and AMC011 SOSIP trimers might be useful platforms for immunogen design to induce consistent and potent NAb responses.

## MATERIALS AND METHODS

### Participants of the Amsterdam Cohort Study on HIV/AIDS.

The AMC009 consensus *env* gene is derived from 5 biological clones isolated from around 2 months post-SC from an HIV-1 subtype B-infected participant in the MSM (men having sex with men) component of the Amsterdam Cohort Studies on HIV/AIDS (ACS) (34, 66). The elite neutralizer H18877, the source of the AMC009 trimer, entered the ACS while HIV-1 negative but seroconverted during active follow-up. Individual H18877 was under observation for more than 5 years without receiving antiretroviral therapy (ART); in that period, his CD4^+^ T-cell count was stable, and he had a detectable viral load. He had no protective HLA type and was homozygous for the CCR5 gene. The AMC011 *env* genes were derived from HIV-1-infected individual D12950, who is enrolled in the intravenous drug user (IDU) component of the ACS and also classified as an elite neutralizer (9, 33). The AMC008 SOSIP trimer is based on an *env* gene that was derived from broad neutralizer H18818 at month 8 post-SC (19, 34).

### Construct design.

*env* genes from five biological clones of individual H18877 (H18877.2m.1B5, H18877.2m.1C3, H18877.2m.1F9, H18877.2m.1G1, and H18877.2m.2B1) from 2 months post-SC were used to derive a consensus sequence, using a cutoff of ≥60% for an amino acid at a specific position (i.e., only amino acids present in 3 of the 5 sequences were included in the consensus sequence). To facilitate affinity purification with the 2G12 bNAb, a PNGS was introduced at position 339 (ENT to NNT). Mutations of the SOSIP.664 design were introduced in the AMC009 consensus sequence, and site-directed mutagenesis was performed to introduce the SOSIP.v4.2 and v.5.2 point mutations using the QuikChange II kit (Stratagene) (18, 19, 29). For some assays, we introduced a D7324 epitope (GSAPTKAKRRVVQREKR) or a His tag (GSGSGGSGHHHHHHHH) sequence at the C terminus of gp41_ECTO_, immediately after residue 664. These analytical trimers are referred to as SOSIP-D7324 and SOSIP-His, respectively, and were used for the following: D7324 capture ELISA (SOSIP-D7324), antitrimer binding antibody ELISA (SOSIP-His), DSC (SOSIP-D7324), and surface plasmon resonance (SPR) (SOSIP-His). The AMC011 SOSIP.v4.2 trimer was constructed in the same way as the AMC009 SOSIP trimer. The consensus sequence is based on three biological clones (33). The v.5.2 mutations were introduced, as described for the AMC009 SOSIP trimer. The AMC008 SOSIP.v4.2 is based on one biological clone (19). To allow the use of the AMC009, AMC011, and AMC008 SOSIP.v5.2 trimers as NAb-depleting reagents in a neutralization assay, a D368R mutation in the CD4-binding site was introduced to prevent binding of the SOSIP trimer to the CD4 receptor on TZM-bl cells. The REJO SOSIP.v4.2 and SHIV162P3 SOSIP.v9 constructs were produced with a D368R mutation for use in NAb depletion and EMPEM analyses, respectively. Similar BG505-based constructs have been described previously (20). The REJO SOSIP.v4.2 D368R trimer was also used for the EMPEM analysis.

### Env protein expression by transient transfection and protein purification.

Codon-optimized *env* genes corresponding to the above-described constructs were obtained from GenScript (Piscataway, NJ) and cloned into the pPPI4 expression vector. All SOSIP trimers used for the experiments were expressed in HEK293F cells by transient transfection and purified by PGT145 affinity chromatography (18). Only SHIV162P3 SOSIP.v9 D368R was purified by PGT151 affinity chromatography. The purification with 2G12 affinity chromatography was made possible for the AMC009 SOSIP trimer with a N339 knock-in but was never used for purification. In some experiments, nontagged proteins were used, while in others the trimers had a C-terminal D7324 or His tag (described above and in the figure and table legends). The tagged and nontagged proteins had generally similar biochemical properties.

### Characterization of SOSIP trimers.

Native-like conformation and the proper furin cleavage were assessed for the SOSIP.v4.2 and v5.2 variants of the AMC009 and AMC011 SOSIP trimers using blue native PAGE (BN-PAGE) and SDS-PAGE gels (18). The same SOSIP proteins were tested in a D324 capture ELISA against a panel of broadly neutralizing and nonneutralizing antibodies (18, 20). The trimers were confirmed to adopt native-like structures, as judged by the exposure of NAb and shielding of non-NAb epitopes. The *T_m_* values for each variant were assessed by DSC and the SOSIP proteins visualized by NS-EM (18).

### Single-particle cryoelectron microscopy.

AMC009 SOSIP.v4.2 trimers were incubated with the PGV04 Fab at a 2-fold molar excess of Fab/protomer overnight at room temperature. The Fab-trimer complex was purified from unbound Fab via a Superose 6 10/300 column (GE Healthcare) in Tris-buffered saline. Fractions containing the complex were pooled and concentrated to 8.5 mg/ml. The following steps were performed as previously described (67). Data collection and processing parameters are reported in Fig. 3. An initial molecular model of the AMC009 SOSIP.v4.2 trimer was generated using the Modeller homology modeling plug-in UCSF Chimera and the soluble JR-FL SOSIP Env structure (PDB entry 5FYK) as a template (39, 68, 69). This model was docked into the EM density map along with the PGV04 Fv (PDB entry 6CRQ) using UCSF Chimera (26, 68). Regions not supported by density were removed, and N-linked glycans were added using Coot (70, 71). The model was iteratively refined into the EM density maps using RosettaRelax and Coot (70–73). Glycan structures were validated using Privateer, and the overall structure was evaluated using EMRinger and MolProbity (74–76). The resolution map (Fig. 3E) was generated in cryoSPARC v2 (77).

### Profiling and site-specific analysis of N-linked glycans.

HILIC-UPLC was used for profiling of N-linked glycans on the AMC009 and AMC011 SOSIP.v4.2 and v5.2 variants. The method was conducted as previously described (47, 49, 78). Site-specific analysis of N-linked glycans and the glycan occupancy at every present PNGS was assessed using mass spectrometry on the v5.2 variants, and the AMC011 SOSIP trimer was D7324 tagged (49). To determine the overall occupancy across two PNGS (N611 and N616), peptides spanning both sites were included. To be included as true positive, b and y ions were required that fragment between the two sites, with one mass signature (+203, +3, or +0) present on each fragment ion.

### Immunizations.

Rabbit immunizations (New Zealand White; 5 animals/group) and blood sampling were carried out under approval number C0048-15 and under subcontract at Covance (Denver, PA, USA). The immunization and bleeding schedules are summarized in Fig. 7A. Animals of the two monovalent groups were immunized with 22 μg of monovalent PGT145-purified trimer (based on peptidic mass) at weeks 0, 4, 20, and 36. Two other groups were immunized with a trivalent combination of SOSIP trimers, following the same schedule as that for the monovalent groups. Animals in the low-dose combination group received 7.3 μg of each SOSIP trimer and 22 μg in the high-dose combination group. All animals received SOSIP.v4.2 trimer variants at weeks 0, 4, and 20 and the SOSIP.v5.2 trimer variant at week 36. All trimers were formulated in 75 U of ISCOMATRIX adjuvant (CSL Ltd., Parkville, VIC, Australia) and rabbits immunized intramuscularly (19, 20, 30, 79). Blood was drawn at weeks 0, 4, 6, 12, 20, 22, 36, and 38. Further analysis was conducted with sera obtained at week 22 and 38, 2 weeks after the third and fourth immunization, respectively, unless stated otherwise.

### Antitrimer binding antibody ELISA.

To assess the binding of serum antibodies from weeks 0, 4, 6, 12, 22, 36, and 38 against the AMC009, AMC011, and AMC008 SOSIP trimers, the purified His-tagged trimers were immobilized on Ni-NTA ELISA plates (Qiagen) (80). Subsequent steps are the same as those for D7324 capture ELISA.

### Neutralization assay.

The autologous and heterologous NAb response of week 22 and week 38 sera was measured in TZM-bl cell neutralization assays (20, 81). Serum IgG was purified and diluted in phosphate-buffered saline (PBS) to the same volume as that of the initial serum (54). The purified IgG was tested in a neutralization assay and treated like serum in terms of input and dilution. In NAb depletion assays, diluted sera were incubated for 45 min with the depleting reagent at a concentration of 80 μg/ml. Depleting reagents included PGT145-purified SOSIP trimers from various isolates, each containing the D368R mutation (18, 80). The test virus was incubated with the mixture of the serum and the depleting reagent for 30 to 60 min. Neutralization was fully depleted with an ID_50_ of <20, indicating all NAbs bound to the depleting reagent.

### AMC009-LAI chimeric clone and neutralization tier categorization.

To test the sensitivity of the parental autologous viruses to bNAbs, infectious molecular clones (IMCs) were constructed using the pLAI backbone, the AMC009 consensus sequence, and the AMC009.1B5 *env* sequence. The ICM based on the consensus sequence of AMC009 was not infectious. However, the biological clone AMC009.1B5 was infectious in the same LAI backbone. AMC009.1B5 differs in three amino acids compared to the AMC009 consensus sequence. The tier categorization of AMC009.1B5 was performed at Duke University Medical Center, and it was classified as a relatively neutralization-resistant (tier 2) virus (Table 1) (82, 83). The parental viruses, based on the AMC011 consensus sequence and AMC008, were classified as tier 1B viruses (19, 50).

### EM-based polyclonal epitope mapping.

Serum IgG was purified from 6 rabbits for which serum NAbs could be depleted by the autologous or heterologous SOSIP protein. The purification was performed as previously described (54). To obtain Fabs, IgG was digested using resin-immobilized papain (50 μl settled resin/mg IgG; Thermo Fisher Scientific) in digestion buffer (Pierce Fab preparation kit and Pierce cysteine-HCl; both from Thermo Fisher Scientific) for 5 h at 37°C at 800 rpm. To remove Fc fragments, the digestion mix was incubated with 0.2 ml of packed protein A agarose (Thermo Fisher Scientific) per initial mg of IgG and incubated at room temperature for 1 h. The Fab-containing sample was buffer exchanged into PBS via ultracentrifugation (10-kDa cutoff; Vivaspin 6). As previously described, Fabs were complexed with SOSIP trimers, size exclusion chromatography purified, and analyzed via NS-EM, and the images were processed (54).

### Statistical analyses.

NAb titers and anti-trimer binding titers between groups were compared using unpaired two-tailed Mann-Whitney *U* test. All statistical analyses were performed in GraphPad Prism 8.

### Data availability.

The AMC009 SOSIP.v4.2 trimer cryo-EM reconstruction and molecular model have been deposited in the Electron Microscopy Data Bank (EMDB) (EMD-21258) and in the Protein Data Bank (PDB entry 6VO3). The cryo-EM maps of the polyclonal sera complex have been deposited in EMDB (EMD-22434 to EMD-22440 and EMD-22442).

## Supplementary Material

Supplemental file 1

Supplemental file 2
