# Supplementary material for: Neutralizing Antibody Responses Induced by HIV-1 Envelope Glycoprotein SOSIP Trimers Derived from Elite Neutralizers
Source: J Virol. 2020 Nov 23;94(24):e01214-20. doi: 10.1128/JVI.01214-20 (PMC7925178; doi:10.1128/JVI.01214-20)
Supplement: Supplemental file 1 [file JVI.01214-20-s0002.pdf]

Table S1. Neutralization of autologous and heterologous viruses by week-22 and -38 sera from rabbits immunized with monovalent or a trivalent combination of subtype B SOSIP trimers. Shown are the serum dilutions at which infection was reduced by 50% (ID_{50}), compared to virus control wells (no serum). The color scheme represents neutralization potency as follows: white: no neutralization (ID_{50} <20) or very weak neutralization (ID_{50} 20-40). Yellow: weak neutralization (ID_{50} 40-100). Orange: moderate neutralization (ID_{50} 100-1000). Red: strong neutralization (ID_{50} >1000). Autologous and heterologous hits against Tier-2 viruses with an ID_{50} ≥100 were verified in one or two independent repeat assays (stars indicate: * one repetition or ** two repetitions), in addition to technical replicates, and the resulting ID_{50} values were averaged and recorded. The neutralization assays were performed at the Academic Medical Center (AMC) or at the Duke University Medical Center (DUMC).
